# Investigating the Mechanism of Action of Diketopiperazines Inhibitors of the *Burkholderia cenocepacia* Quorum Sensing Synthase CepI: A Site-Directed Mutagenesis Study

**DOI:** 10.3389/fphar.2018.00836

**Published:** 2018-07-31

**Authors:** Silvia Buroni, Viola C. Scoffone, Marco Fumagalli, Vadim Makarov, Maddalena Cagnone, Gabriele Trespidi, Edda De Rossi, Federico Forneris, Giovanna Riccardi, Laurent R. Chiarelli

**Affiliations:** ^1^Department of Biology and Biotechnology “Lazzaro Spallanzani”, University of Pavia, Pavia, Italy; ^2^Bach Institute of Biochemistry, Research Center of Biotechnology, Russian Academy of Sciences, Moscow, Russia; ^3^Department of Molecular Medicine, University of Pavia, Pavia, Italy

**Keywords:** quorum sensing, Acyl Homoserine Lactone synthase, synthase inhibitors, *Burkholderia cenocepacia*, homology model, site-directed mutagenesis

## Abstract

Quorum sensing (QS) is a bacterial intercellular communication process which controls the production of major virulence factors, such as proteases, siderophores, and toxins, as well as biofilm formation. Since the inhibition of this pathway reduces bacterial virulence, QS is considered a valuable candidate drug target, particularly for the treatment of opportunistic infections, such as those caused by *Burkholderia cenocepacia* in cystic fibrosis patients. Diketopiperazine inhibitors of the acyl homoserine lactone synthase CepI have been recently described. These compounds are able to impair the ability of *B. cenocepacia* to produce proteases, siderophores, and to form biofilm, being also active in a *Caenorhabditis elegans* infection model. However, the precise mechanism of action of the compounds, as well as their effect on the cell metabolism, fundamental for candidate drug optimization, are still not completely defined. Here, we performed a proteomic analysis of *B. cenocepacia* cells treated with one of these inhibitors, and compared it with a *cepI* deleted strain. Our results demonstrate that the effects of the compound are similar to the deletion of *cepI*, clearly confirming that these molecules function as inhibitors of the acyl homoserine lactone synthase. Moreover, to deepen our knowledge about the binding mechanisms of the compound to CepI, we exploited previously published *in silico* structural insights about this enzyme structure and validated different candidate binding pockets on the enzyme surface using site-directed mutagenesis and biochemical analyses. Our experiments identified a region near the predicted *S*-adenosylmethionine binding site critically involved in interactions with the inhibitor. These results could be useful for future structure-based optimization of these CepI inhibitors.

## Introduction

Quorum sensing (QS) is a bacterial intercellular communication process, which relies on the synthesis and secretion of signal molecules (Sokol et al., 2007; Udine et al., 2013). The binding of these molecules to specific effectors mediates the regulation of the expression of major virulence factors such as proteases, siderophores, and toxins (Tomlin et al., 2005) and enhances the ability of bacteria to form biofilm. This last characteristic greatly impairs the diffusion of antibiotics enhancing resistance to antibacterial compounds. In this way, QS can be considered a good candidate drug target, as the interference with this pathway makes bacteria less virulent (Rasko and Sperandio, 2010).

This strategy could be particularly useful for the treatment of opportunistic pathogens, such as *Burkholderia cenocepacia*, a Gram negative bacterium which colonizes the lung of cystic fibrosis patients (Drevinek and Mahenthiralingam, 2010). These infections are particularly dangerous. This is due to the high rate of resistance to antibiotics caused by enzymatic inactivation, modification of target, poor cell wall permeability, and the presence of many efflux pumps (Scoffone et al., 2017). Thus, the identification of new compounds able to inhibit *B. cenocepacia* growth, as well as of new drug targets, is a prominent question.

*B. cenocepacia* J2315 possesses four QS systems composed by a synthase (I) and a receptor (R): CepIR, CciIR, the *Burkholderia* Diffusible Signal Factor (BDSF)-based system RpfF_BC,_ and the recently discovered non-ribosomal peptide synthetase-like cluster *ham* (Coenye, 2010; Spadaro et al., 2016; Jenul et al., 2018). The characterization of *B. cenocepacia* mutants lacking the synthases CepI and/or CciI and RpfF_BC_ demonstrated an involvement of CepI in biofilm formation, protease production, and virulence, as well as an interplay among the Acyl Homoserine Lactone (AHL) systems CepIR and CciIR and the BDSF-based system (Udine et al., 2013).

We recently identified new diketopiperazine molecules, able to inhibit CepI *in vitro*, impairing the ability of *B. cenocepacia* to produce proteases, siderophores, and to form biofilm (Scoffone et al., 2016). These molecules did not possess any antimicrobial activity, nevertheless their administration significantly increased the survival of *Caenorhabditis elegans* nematodes infected with *B. cenocepacia*, suggesting that the virulence of the strain could be attenuated *in vivo*. All these data suggest the possibility of a combined treatment by using CepI inhibitors with antimicrobials, to improve the therapeutic strategies available against *B. cenocepacia* (Scoffone et al., 2016).

**FIGURE 1:**
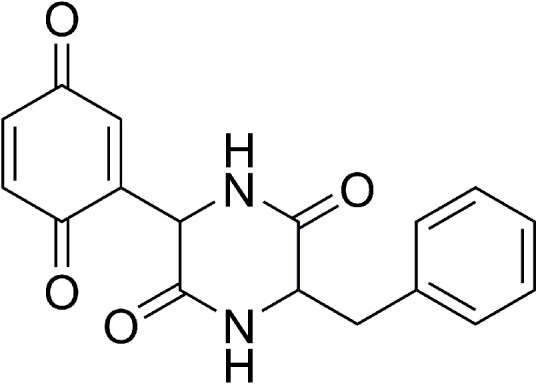
Chemical structure of the (3S)-3-Benzyl-6-(3,6-dioxocyclohexa-1,4-dien-1-yl)piperazine-2,5-dione (8b) CepI inhibitor.

The current lack of molecular structure data on CepI prevents the possibility of 3D structure-assisted optimization studies of these new inhibitors. In our previous work, we generated a CepI homology model, and used it to perform *in silico* docking analyses of the diketopiperazine inhibitor 8b (**Figure 1**) (“Supplementary Materials and Methods”). Using this approach, we identified multiple candidate binding sites, localized far from the enzyme catalytic site, but in regions possibly still implicated in substrate recognition and catalysis (Scoffone et al., 2016).

Here, we confirmed that the cellular effects of 8b are indeed related to the inhibition of CepI, by analyzing the proteome of *B. cenocepacia* cells treated with the compound, and compared with that of the knock-out *cepI* strain. Moreover, we exploited a site-directed mutagenesis strategy to better define the crucial amino acid residues responsible for catalysis and recognition of the 8b inhibitor. Taken together, our results suggest a possible mechanism of CepI inhibition by the 8b compound through perturbations of a flexible loop involved in recognition and stabilization of the *S*-adenosylmethionine substrate, facilitating future drug discovery approaches based on the 8b chemical scaffold.

## Materials and Methods

### Site-Directed Mutagenesis

Plasmid pETSUMO-CepI (Scoffone et al., 2016) was used as template for each PCR mutagenesis experiment to generate amino acid substitutions using the primers listed in Supplementary Table S1. The site-directed mutagenesis was carried out as previously described in the PCR-based method (Bachman, 2013) using HotStar HiFidelity Polymerase (Qiagen) according to manufacturer’s instructions.

### Proteins Purification and Characterization

Wild type and mutant CepI were expressed in *Escherichia coli* BL21(DE3) cells and purified as previously described (Scoffone et al., 2016).

Far-UV circular dichroism (CD) measurements were performed with a JascoJ-700 spectropolarimeter (Jasco-Europe, Cremella, Italy) using a 1 mm path cell. Scans were conducted between 190 and 250 nm at a speed of 50 nm/min with a spectral band width of 2 nm and a sensitivity of 20 mdeg. CD spectrum measurements were performed at 25°C in 50 mM sodium phosphate pH 8.0, 50 mM KCl, and represent the average of 10 scans. The protein concentration was 4–5 μM. Spectra were analyzed using the DichroWeb online platform (Whitmore and Wallace, 2008).

CepI activity was determined according to Christensen et al. (2013). Reaction mixtures contained 50 mM 4-(2-hydroxyethyl)-1-piperazineethanesulfonic acid (HEPES) pH 7.5, 0.005% Nonidet P-40, 0.13 mM 2,6-dichlorophenylindophenol (DCPIP), 70 μM Octanoyl-ACP (C8-ACP, prepared as reported previously) (Quadri et al., 1998; Cronan and Thomas, 2009), 40 μM *S*-adenosyl methionine (SAM), 4 μM CepI.

Steady-state kinetic parameters were determined by assaying the enzymes at eight different concentrations of substrates, using the Michaelis–Menten equation and Origin eight software.

IC_50_ was determined measuring the enzyme activity in presence of different concentrations of compound, and values determined with the following equation (Copeland, 2000), where A_[I]_ is the enzyme activity at inhibitor concentration [I] and A_[0]_ is the enzyme activity without inhibitor.

$${\text{A}}_{\left[\text{I}\right]}={\text{A}}_{\left[0\right]}\times (1-\frac{\left[\text{I}\right]}{[\text{I}]+{\text{IC}}_{50}})$$

### Proteomics Analysis

*Burkholderia cenocepacia* Δ*cepI* (Udine et al., 2013) and J2315 (with or without 25 μM of 8b compound) were grown in 10 ml of LB medium until OD_600__nm_ > 2. Cells were then harvested, resuspended in 0.2 ml of Tris-HCl 50 mM, pH 7.5, disrupted by sonication, and centrifuged at 12,000 rpm for 1 h at 4°C.

The amount of proteins present in the supernatant was quantified by bicinchoninic acid method (Smith et al., 1985), then 300 μg were precipitated with 10% (v/v) trichloroacetic acid. The obtained protein pellet was dissolved in 125 μL of rehydration buffer (8 M urea, 4% CHAPS (w/v), 65 mM DTE, 0.8% carrier ampholytes (v/v) and 0.5% bromophenol blue) and loaded onto 7 cm IPG strips, with nonlinear pH 3–10 gradient range (GE Healthcare), and strips rehydrated for 1 h at 20°C. The first-dimensional IEF was carried out at 15°C using an Ettan IPGphor system (GE Healthcare), by applying 30 V for 8 h, 120 V for 1 h, 500 V for 0.5 h, 1000 V for 0.5 h and 5000 V for 6 h, for a total of 29–30 kVh. The focused IPG strips were subjected to reduction/alkylation steps then loaded onto an 8 × 6 cm slabs, 12.5% SDS polyacrylamide gels. The 2-DE gels were stained with “Blue silver” (colloidal Coomassie G-250 staining), according to Candiano et al. (2004). Digital images of stained gels were acquired using VersaDoc Imaging Model 3000 (BioRad) and then subjected to quali/quantitative analysis using the PD Quest (BioRad) version 8.0.1 software. Scanned images were filtered and smoothed to remove background noise, vertical/horizontal streaking, gel artifacts and then normalized to eliminate the variability of each sample. The software then determined the amount of spots present and calculated their intensity by applying the following algorithm: peak value (ODs/image units) ^∗^σ_*x*_^∗^σ_y_ (standard deviations in x and y).

For protein identification, the selected spots were carefully excised from the gel, washed twice with 100 mM ammonium bicarbonate buffer pH 7.8, 50% acetonitrile (ACN) and kept under stirring overnight, until complete destaining. After dehydration, gels were rehydrated by addition of 50 μL of 100 mM ammonium bicarbonate buffer pH 7.8, containing 20 ng/μL sequencing grade trypsin (Promega, Madison, WI, United States) and digestion was performed overnight at 37°C. The resulting peptides were extracted sequentially from gel matrix by treatment with 50 μL of 50% ACN in water, 5% trifluoroacetic acid and finally with 50 μL of 100% ACN. Each extraction involved 15 min of stirring followed by centrifugation and removal of the supernatant. The original supernatant and those obtained from sequential extractions were pooled, dried and stored at −80°C until mass spectrometric analysis. At the moment of use, the peptide mixture was solubilized in 100 μL of 0.1% formic acid (FA) for MS analyses.

The analyses were carried out on an LC-MS (Thermo Finnigan, San Jose, CA, United States) system consisting of a thermostated column oven Surveyor autosampler controlled at 25°C; a quaternary gradient Surveyor MS pump equipped with an UV/V is detector and an Ion Trap (LCQ Fleet^TM^) mass spectrometer with electrospray ionization ion source controlled by Xcalibur software 2.0.7. Analytes were separated by RP-HPLC on a Jupiter (Phenomenex, Torrance, CA, United States) C_18_ column (150 × 2 mm, 4 μm, 90 Å particle size) using a linear gradient (2–60% solvent B in 60 min) in which solvent A consisted of 0.1% aqueous FA and solvent B of ACN containing 0.1% FA. Flow-rate was 0.2 mL/min. Mass spectra were generated in positive ion mode under constant instrumental conditions: source voltage 5.0 kV, capillary voltage 46 V, sheath gas flow 20 (arbitrary units), auxiliary gas flow 10 (arbitrary units), sweep gas flow 1 (arbitrary units), capillary temperature 210°C, tube lens voltage 105 V. MS/MS spectra, obtained by CID studies in the linear ion trap, were performed with an isolation width of 3 Da *m/z*, the activation amplitude was 35% of ejection RF amplitude that corresponds to 1.58 V.

Data processing was performed using Peaks Studio version 4.5 software. The mass list was searched against the SwissProt protein and *B. cenocepacia* databases, under continued mode (MS plus MS/MS) with the following parameters: trypsin specificity; five missed cleavages; peptide tolerance at 0.2 Da and MS/MS tolerance at 0.25 Da; peptide charge 1, 2, 3+ and experimental mass values: monoisotopic.

## Results

### Diketopiperazine 8b Treatment and *cepI* Knock-Out Have the Same Phenotypic Effects

In a previous study, we identified a diketopiperazine molecule active against the QS synthase CepI, and impairing the production of virulence factors in *B. cenocepacia* J2315, as well as the ability of the bacterium to form biofilm (Scoffone et al., 2016).

**FIGURE 2:**
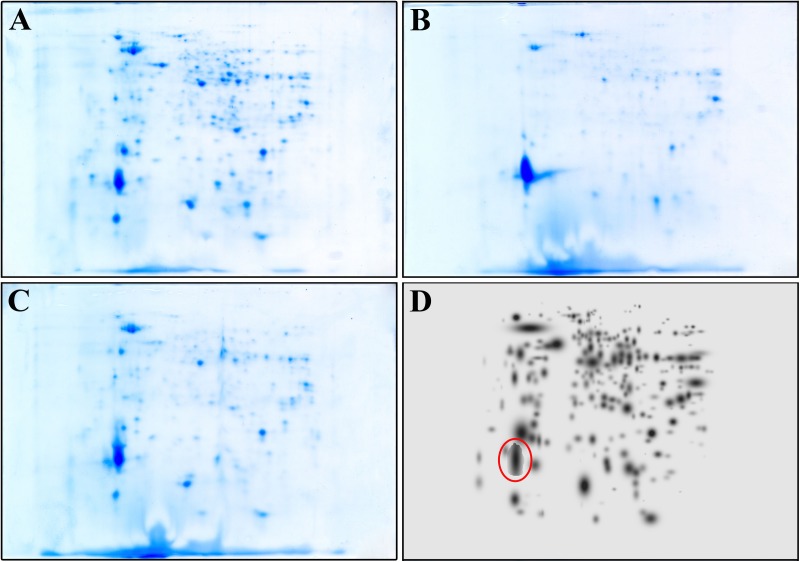
Representative 2-DE map of *B. cenocepacia* J2315 untreated **(A)**, treated with 8b 25 μM **(B)** and of Δ*cepI* knock-out strain **(C)**. **(D)** represents the High Master Gel, a virtual image resulting from the correlation of the master gels of each sample group. Red circle highlight the spot corresponding to the giant cable pilus protein.

To further confirm that the compound acts also intracellularly inhibiting the CepI synthase, we compared the proteomes of the wild type *B. cenocepacia* J2315 untreated and treated with 25 μM 8b, with that of the Δ*cepI* knock-out strain. To identify the bacterial proteins differentially expressed, parallel 2-DE analyses of protein extracts from each cell lysate were performed in triplicate. **Figures 2A–C** shows the representative maps of the three samples.

A match set was then created to compare the gels of each replicate and to match the spots present, and used to get a synthetic image (Master Gel) containing qualitative and quantitative data relative to all spots. The Master Gels from each group (*B. cenocepacia* J2315 untreated and treated, and Δ*cepI*) were matched to create a virtual image (High Master Gel, **Figure 2D**), containing all the common and uncommon spots. This higher match set allowed to determine the presence or absence of spots and the intensity values of common ones; typically, a mean of 240 protein spots were detected in each gel. The comparison of the 2-DE patterns revealed qualitative and quantitative differences between the three groups considered. In terms of presence/absence, the majority of spots were in common between *B. cenocepacia* J2315 cells treated with 8b and Δc*epI* cells, while protein spots showed different density compared with the untreated J2315. Among them, one protein spot (marked in red in **Figure 2D**) showed higher density in 8b treated cells and Δ*cepI* profiles, and was excised from the gel for LC-MS/MS identification. This spot was found to correspond to the giant cable pilus protein CblA (Supplementary Table S2), which is known to contribute to *B. cenocepacia* virulence, being involved in persistence *in vivo* (Goldberg et al., 2011) and to adherence to respiratory epithelia (Sajjan et al., 2000). Moreover, the *cblA* gene is a useful marker for the *B. cenocepacia* ET12 lineage and has been linked with both pathogenicity and epidemic behavior (Mahenthiralingam et al., 2000). Thus, our data demonstrate that 8b mimics the effects of the deletion of *cepI*, confirming that the effects of the compound clearly rely on CepI inhibition.

### Identification of the Diketopiperazine 8b Binding Site on CepI Enzyme

**FIGURE 3:**
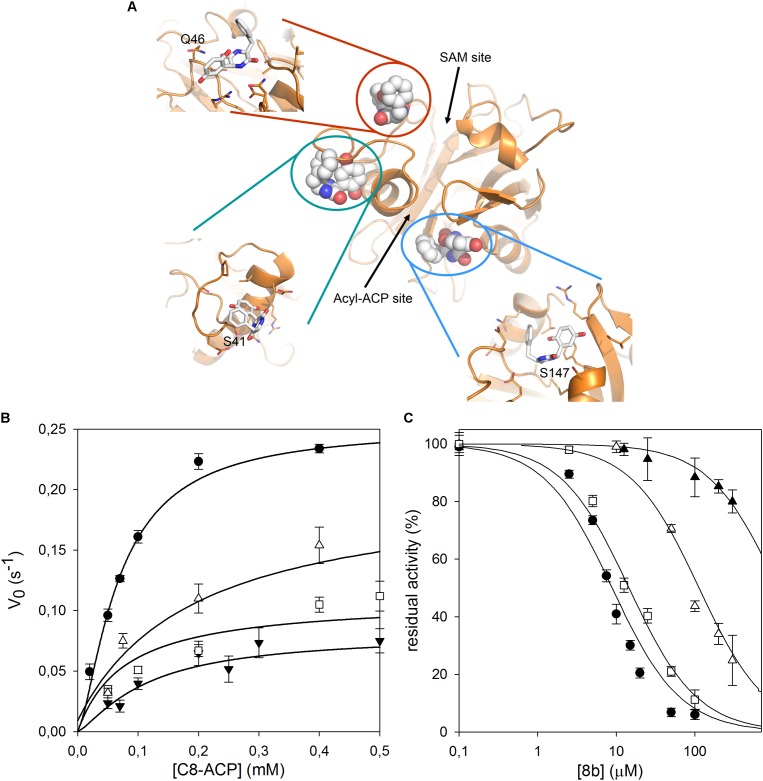
Identification of the 8b binding site on CepI. **(A)** Cartoon representation of the CepI homology model showing three candidate binding sites, according to extensive *in silico* docking experiments using the 8b compound performed as described in (Scoffone et al., 2016) and in “Supplementary Materials and Methods.” We selected three amino acid residues S41, Q46, and S147, belonging to each pocket, for mutagenesis study. **(B)** Steady state kinetic analysis of the two active CepI mutants as a function of C8-ACP. **(C)** IC_50_ determination of 8b against CepI enzymes. 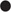: wild type; 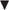: S41R; 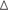: S41A; 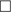: Q46R. The bars represent the standard deviation. Median values of three biological replicates are reported.

As no structural data on *B. cenocepacia* CepI are available, to improve the possibility of 3D structure-assisted optimization of the non-competitive inhibitor 8b, we previously generated a homology model of the enzyme and performed *in silico* docking studies, which enabled the identification of multiple candidate binding sites for the inhibitor (Scoffone et al., 2016 and “Supplementary Materials and Methods”). In particular, 8b could be docked with high score in at least three different pockets on the enzyme surface (**Figure 3A**). To understand if the inhibitor effectively binds to these sites, we therefore generated three different CepI mutants: S41R, Q46R, and S147R.

All mutants could be expressed and purified to homogeneity using the same procedure adopted for the wild type (Scoffone et al., 2016). Although these mutants were obtained with yields generally lower than wild type CepI, all proteins variants were found soluble. Moreover, CD spectra of the mutants did not show significant differences with that of the wild type, thus confirming that the introduced mutations did not affect the correct fold of the protein (Supplementary Figure S1).

Among these mutants, our biochemical experiments revealed that S147R was completely inactive (**Table 1**). To ascertain if the lack of activity was due to the introduction of a bulky and positively charged residue, we produced a new S147L mutant. However, this mutant was also inactive (**Table 1**), suggesting an essential role for the area around the amino acid 147 in the catalysis.

**Table 1:** Enzymatic characterization of CepI mutants.

Enzyme	*V*_max_ (s^−1^)	*K*_*m*_ (mM)	compound 8b IC_50_ (mM)
Wild type	0.25 ± 0.009	0.068 ± 0.005	0.0072 ± 0.0002
R24Q	0.16 ± 0.020	0.074 ± 0.026	0.147 ± 0.017
E29Q	n. a.^a^	n. a.	n. a.
E40Q	0.09 ± 0.003	0.105 ± 0.001	0.053 ± 0.0026
S41A	0.16 ± 0.007	0.081 ± 0.006	0.113 ± 0.011
S41R	0.08 ± 0.010	0.113 ± 0.016	1.070 ± 0.075
Q46R	0.18 ± 0.024	0.078 ± 0.013	0.0192 ± 0.0035
S147L	n. a.	n. a.	n. a.
S147R	n. a.	n. a.	n. a.

*^a^n.a. not active.*

By contrast, the S41R and Q46R mutants did not show severe perturbations in their kinetic properties, showing *K*_m_ values unchanged respect to the wild type, and *k*_cat_ values two to three fold reduced (**Figure 3B** and **Table 1**). To understand whether any of these mutated residues could be involved in the binding of the inhibitor, we measured the IC_50_ of compound 8b toward each mutant enzyme. While Q46R was comparable to wild type CepI, S41R was practically insensitive to the compound (IC_50_ > 1 mM) (**Figure 3C** and **Table 1**), strongly suggesting an involvement of this residue in the mechanism of action of 8b. However, to ascertain if the high IC_50_ of the compound toward the S41R mutant was due to perturbations introduced by the bulky and positively charged arginine rather than to the lack of interaction with the serine, a more conservative mutant S41A was generated. This new mutant was still less sensitive to 8b, showing an IC_50_ 15-fold higher than that of the wild type (**Figure 3C** and **Table 1**), thus confirming a direct involvement of Ser 41 in inhibitor binding.

**FIGURE 4:**
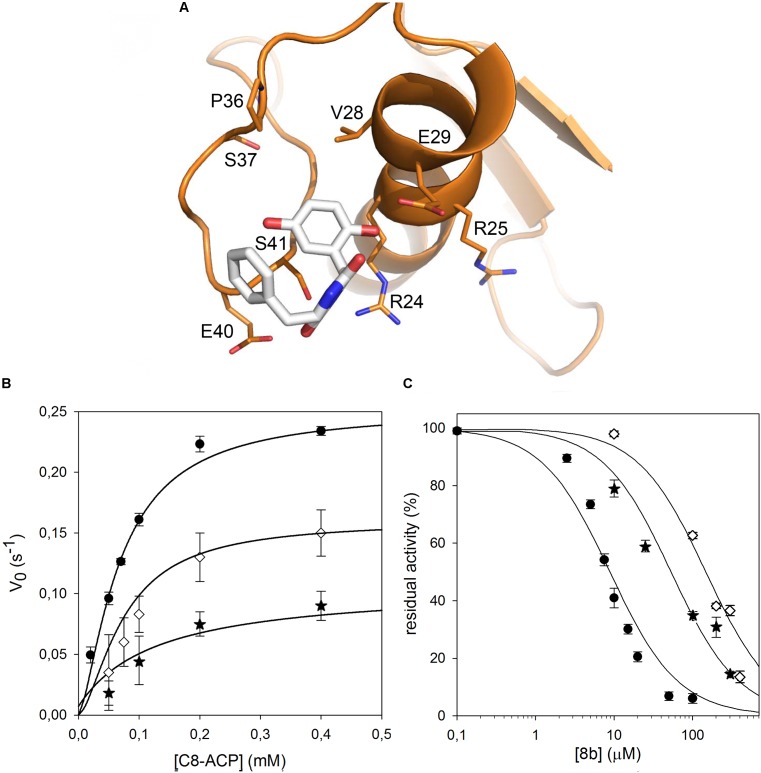
Identification of the CepI amino acid residues involved in 8b binding. **(A)** Cartoon representation of the CepI homology model showing the possible interaction of 8b with the residues in proximity to the serine 41. The three amino acid R24, E29, and E40 were selected for mutagenesis. **(B)** Steady state kinetic analysis of the two active CepI mutants as a function of C8-ACP. **(C)** IC_50_ determination of 8b against CepI enzymes. 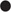: wild type; 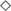: R24Q; 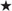: E40Q. The bars represent the standard deviation. Median values of three biological replicates are reported.

We therefore decided to expand our investigation to other amino acids belonging to the surface of this pocket region. This region is proximate to a loop that adopts a variety of conformations in homologous AHL synthases, with possible implications in SAM binding. In particular, we studied the role of R24, E29, and E40, predicted to constitute contact platform for the compound or for serine 41. Each of these residues was mutated into a glutamine (**Figure 4A**). Once again all these mutants did not show significant alteration of the *K*_m_ values; a small reduction was detected in their *k*_cat_, with the exception of the E29Q which however displayed negligible enzymatic activity (**Figure 4B** and **Table 1**). Also in this case, the CD spectrum of E29Q did not show differences with respect to the wild type, thus excluding that the lack of catalytic activity is due to a misfolding of the mutant (Supplementary Figure S1).

By contrast, all the active mutants were significantly less inhibited by the 8b compound, showing IC_50_ values 10–15 fold higher respect to the wild type (**Figure 4C** and **Table 1**).

These results confirmed that CepI residues located in this region have a direct role in the binding and in the mechanism of inhibition of 8b against CepI, and experimentally support our previous hypotheses driven by *in silico* analyses.

## Discussion

Quorum sensing inhibitors appear as very promising potentiators of the classical antibiotic therapy to treat dangerous infections such as those caused by *B. cenocepacia*, for which a lack of new solutions is massively reported (Horsley et al., 2011; Regan and Bhatt, 2016; Scoffone et al., 2017). Searching in PubMed for “quorum sensing inhibitors” retrieves 51 results in the first 5 months of 2018, which shows the increasing interest in alternative routes to counteract the emergence of resistance among bacteria such as *Proteus mirabilis* (Yu et al., 2018), *Salmonella* (de Almeida et al., 2018), *Pseudomonas aeruginosa* (Almohaywi et al., 2018), *Staphylococcus aureus* (Karathanasi et al., 2018).

We recently reported the discovery of diketopiperazines as inhibitors of the QS synthase CepI of *B. cenocepacia*. Beside the *in vitro* effect on the purified protein, these inhibitors showed interesting *in vivo* phenotypes, being able to decrease the production of proteases, siderophores, and lowering the ability of *B. cenocepacia* to form biofilm. Moreover, they increased the survival of infected *C. elegans* (Scoffone et al., 2016).

Here, we further confirmed that the compound acts also intracellularly by comparing the proteomes of the wild type *B. cenocepacia* J2315 untreated and treated with 25 μM 8b, with that of the Δ*cepI* knock-out strain. Indeed, these last two cultures showed the same proteomic profile: in our experimental conditions, only the over production of the giant cable pilus protein CblA was detected. Cable pilus is a well known virulence factor, which together with the 22-kDa pilus-associated adhesin is mainly involved in binding of the host cells and, particularly, in the transmigration across the epithelium (Urban et al., 2005). However, no relationship between CepI and CblA has been reported so far. Thus, this result is quite unexpected and why the lack or inhibition of the QS synthase should enhance the expression of this virulence trait of *B. cenocepacia* is an intriguing open question which is worth investigating deeper.

In our previous work, we computationally generated an homology model of *B. cenocepacia* CepI and performed *in silico* docking procedures, which enabled the identification of three candidate binding sites for the most promising diketopiperazine compound 8b (Scoffone et al., 2016). To confirm the involvement of these pockets into 8b binding, we initially designed three different CepI mutants: S41R, Q46R, and S147R. As expected from homology model analysis, our biochemical experiments revealed that the area surrounding the amino acid 147 is likely involved in catalysis due to strong proximity of this residue to the main access cavity hosting the acyl-ACP substrates (**Figure 3A**). Intriguingly, mutant S41R was practically insensitive to the 8b compound, suggesting that the pocket around this residue may form the binding site of the compound. Thus, to confirm this, three other mutants were generated by modifying the charged residues (R24, E29, and E40) around S41 into the polar, uncharged glutamine. Mutation E29Q abolished CepI enzymatic activity, suggesting roles for this residue in catalysis despite its distance from the enzyme’s catalytic site. The other two mutants did not show significant alteration of the kinetic properties, nevertheless they were significantly less susceptible to inhibition by the 8b compound, confirming the mechanism of action and the involvement of the pocket in 8b binding.

**FIGURE 5:**
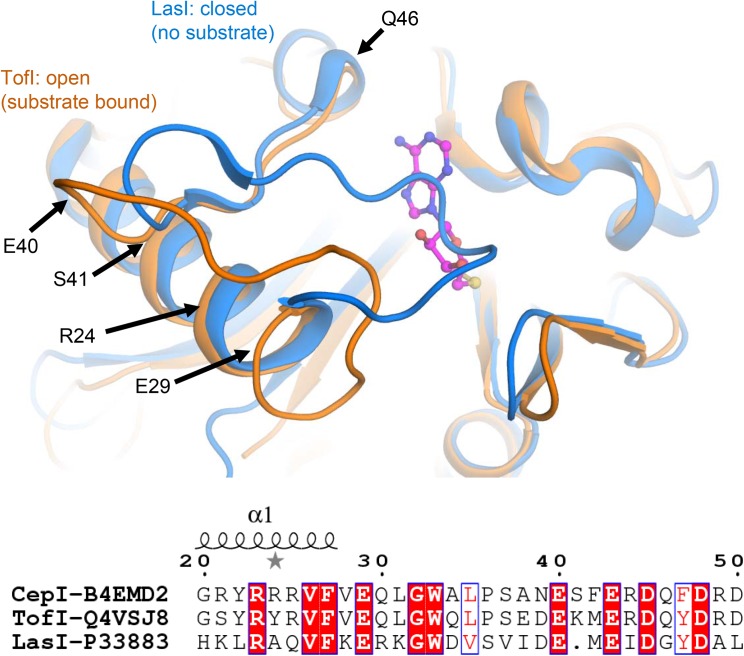
Identification of CepI mutated residues based on structural similarity with the crystal structures of homologous AHL synthases TofI (Chung et al., 2011) (orange) and LasI (Gould et al., 2004) (blue). The position of the SAM substrate, derived from the crystal structure of TofI in complex with an analog, is shown with magenta ball-and-sticks. The position of residues 24, 29, 40, 41, and 46 are shown with black arrows. The amino acid composition of this region is partially conserved among AHL synthases, as highlighted in the bottom panel, in which the fully conserved residues are evidenced, while conservative changes are in red.

Comparisons with homologous AHL synthases TofI (Chung et al., 2011) and LasI (Gould et al., 2004) structures indicate that CepI loop comprising neighboring residues 30–41 may be involved in conformational changes associated to interactions with the SAM substrate. These conformational changes may involve partial unfolding of the last helical turn comprising the fully conserved E29 residue. In this respect, isosteric alterations of the negatively charged character of this amino acid may result in increased structural instability and, possibly, interfere with the productive conformational changes necessary for SAM substrate binding (**Figure 5**).

**FIGURE 6:**
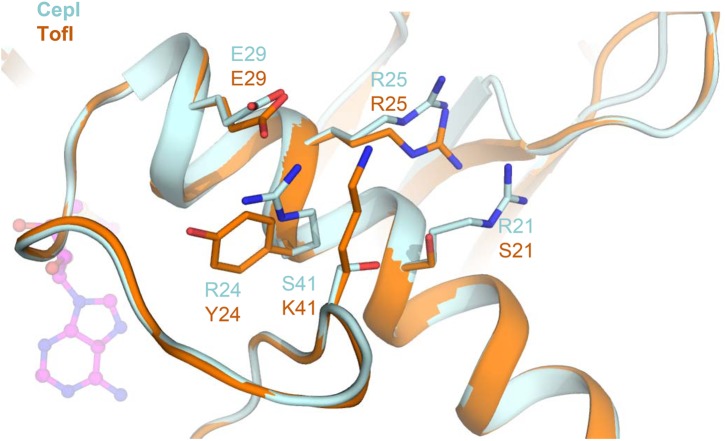
Comparison of the amino acid network surrounding residue 41 in CepI and TofI. Shown is a superposition of the CepI homology model (light blue) with TofI crystal structure (Chung et al., 2011) (orange). Amino acid side chains are shown as sticks and labeled according to their numbering in both CepI and TofI. The position of the SAM substrate analog, as derived from TofI crystal structure, is shown as magenta ball-and-stick.

Residue S41, identified as the most susceptible to compound 8b inhibition, is located at the C-terminal hinge of the flexible loop possibly subject to conformational changes upon SAM binding (**Figure 6**). Introduction of bulky, positively charged residues at this site (such as in the S41R mutant) may introduce strong repulsion due to interference with neighboring Arg21 and Arg25 residues and may possibly explain the dramatic variation observed in IC_50_ values (**Table 1**). Notably, homologous TofI possesses a Lys residue at this site, but at the same time its corresponding residue at position 21 is not an Arg, but a smaller Ser residue (**Figure 6**).

Together these results support our previous finding and confirm that compound 8b acts intracellularly by inhibiting CepI activity, thus interfering with the production of the QS signal molecule C8-AHL. Moreover, the identification of the pocket involved in inhibitory mechanism of 8b provides new information on the binding of the compound with CepI, which will be useful for a structure-based optimization of the diketopiperazine inhibitors.

## Author Contributions

SB, VS, and LC conceived and directed the project. SB, FF, and LC designed the experiments. VS, MF, VM, GT, and SB, carried out the experiments. SB, EDR, FF, GR, and LC conducted the data analysis and interpreted the results. SB, VS, FF, and LC wrote the manuscript. All the authors read and approved the final version of the manuscript.

## Conflict of Interest Statement

The authors declare that the research was conducted in the absence of any commercial or financial relationships that could be construed as a potential conflict of interest.
