# Supplementary material for: Investigating the Mechanism of Action of Diketopiperazines Inhibitors of the Burkholderia cenocepacia Quorum Sensing Synthase CepI: A Site-Directed Mutagenesis Study
Source: Front Pharmacol. 2018 Jul 31;9:836. doi: 10.3389/fphar.2018.00836 (PMC6079302; doi:10.3389/fphar.2018.00836)
Supplement: Supplementary file 1 [file Data_Sheet_1.DOCX]

Supplementary material

**Investigating the mechanism of action of diketopiperazines inhibitors of the *Burkholderia cenocepacia* quorum sensing synthase CepI: a site directed mutagenesis study.**

**Silvia Buroni, Viola Camilla Scoffone, Marco Fumagalli, Vadim Makarov, Gabriele Trespidi, Edda De Rossi, Federico Forneris, Giovanna Riccardi, and Laurent Roberto Chiarelli**

***Correspondence:** Dr. Laurent R. Chiarelli: laurent.chiarelli@unipv.it

**Supplementary Methods**

*CepI homology model and structural analyses* – A CepI homology model was generated computationally as described in (Scoffone et al., 2016). Briefly, candidate structural homologs were shortlisted using multiple primary sequence alignments using HHPRED^S1^. The ten top scoring structural models were fed as structural templates for homology model generation into MODELLER^S2^. The model was further optimized by geometry idealization using PHENIX^S3^ . Final model quality was assessed using PROCHECK^S4^ and the Qmean server^S5^. Superpositions were done using the secondary structure matching (SSM) protocols available in COOT^S6^. Structural figures were generated using PyMol^S7^.

*Docking analysis of 8b –* Docking data were previously generated in (Scoffone et al., 2016) using unconstrained ligand exploration using the SWISSDOCK^S8^ and PELE^S9^docking servers, followed by parallel cycles of ligand binding minimization and refinement in PELE^S9^ (12 hours runs on a 64-CPU server) of the most stable candidate binding sites selected based on predicted stabilization energy and residual ligand mobility. These conformations were grouped into two subsets based on (1) their proximity to structural elements critical for substrate recognition or (2) elsewhere on the CepI surface. In this work, we intentionally excluded docking results from subset 2 and focused on results from subset 1, composed of the three candidate binding sites shown in Figure 3 (for each site, the most stable binding conformation of the 8b compound predicted from docking experiments is shown).

**Table S1**. Oligonucleotides used for site directed mutagenesis. The underlined sequences indicate the mutated bases.

| CepIR24Qfor | 5’-CTCGGGCGCTATCGGC**AG**CGCGTGTTCGTCGAGC-3’ |
| --- | --- |
| CepIR24Qrev | 5’-GCTCGACGAACACGCG**CT**GCCGATAGCGCCCGAG-3’ |
| CepIE29Qfor | 5’-CGCGTGTTCGTC**CAG**CAGCTCGGTTGGGCG-3’ |
| CepIE29Qrev | 5’-CGCCCAACCGAGCTG**CTG**GACGAACACGCG-3’ |
| CepIE40Qfor | 5’-CCCGTCGGCGAAC**C**AAAGTTTCGAGCGTGACC-3’ |
| CepIE40Qrev | 5’-GGTCACGCTCGAAACTTT**G**GTTCGCCGACGGG-3’ |
| CepIS41Afor | 5’-CCCGTCGGCGAACGAA**GC**TTTCGAGCGTGACC-3’ |
| CepIS41Arev | 5’-GGTCACGCTCGAAA**GC**TTCGTTCGCCGACGGG-3’ |
| CepIS41Rfor | 5’-CCCGTCGGCGAACGAA**C**GTTTCGAGCGTGACC-3’ |
| CepIS41Rrev | 5’-GGTCACGCTCGAAAC**G**TTCGTTCGCCGACGGG-3’ |
| CepIQ46Rfor | 5’-GAAAGTTTCGAGCGTGACC**G**GTTCGATCGCGACGATACC-3’ |
| CepIQ46Rrev | 5’-GGTATCGTCGCGATCGAAC**C**GGTCACGCTCGAAACTTTC-3’ |
| CepIS147Rfor | 5’-GATCGGCGTGACGTTCGCG**C**GCATGGAGCGGCTGTTCCG-3’ |
| CepIS147Rrev | 5’-CGGAACAGCCGCTCCATGC**G**CGCGAACGTCACGCCGATC-3’ |
| CepIS147Lfor | 5’-GATCGGCGTGACGTTCGCG**CT**CATGGAGCGGCTGTTCCGC-3’ |
| CepIS147Lrev | 5’-GCGGAACAGCCGCTCCATG**AG**CGCGAACGTCACGCCGATC-3’ |

**Table S2**. Proteins identified with additional information such as the primary sequence of peptides.

|  | | | | | |
| --- | --- | --- | --- | --- | --- |
| Accession | Mass | Score (%) | Description | z | Peptides |
| tr\|B4ELG0\|B4ELG0_BURCJ | 17,048 | 99 | Giant cable pilus | 1 | IFTNDK |
|  |  |  |  | 1 | LATAPALK |
|  |  |  |  | 1 | DLQIR |
|  |  |  |  | 2 | NQTSPGAAEIPLSVK |
|  |  |  |  | 2 | LGETELTTTAATLK |
|  |  |  |  | 2 | KVEAVTASGSYQGLVSVIVTQSAASGS |
|  |  |  |  | 2 | TAELFPGELAQGSNVLALSIGQK |


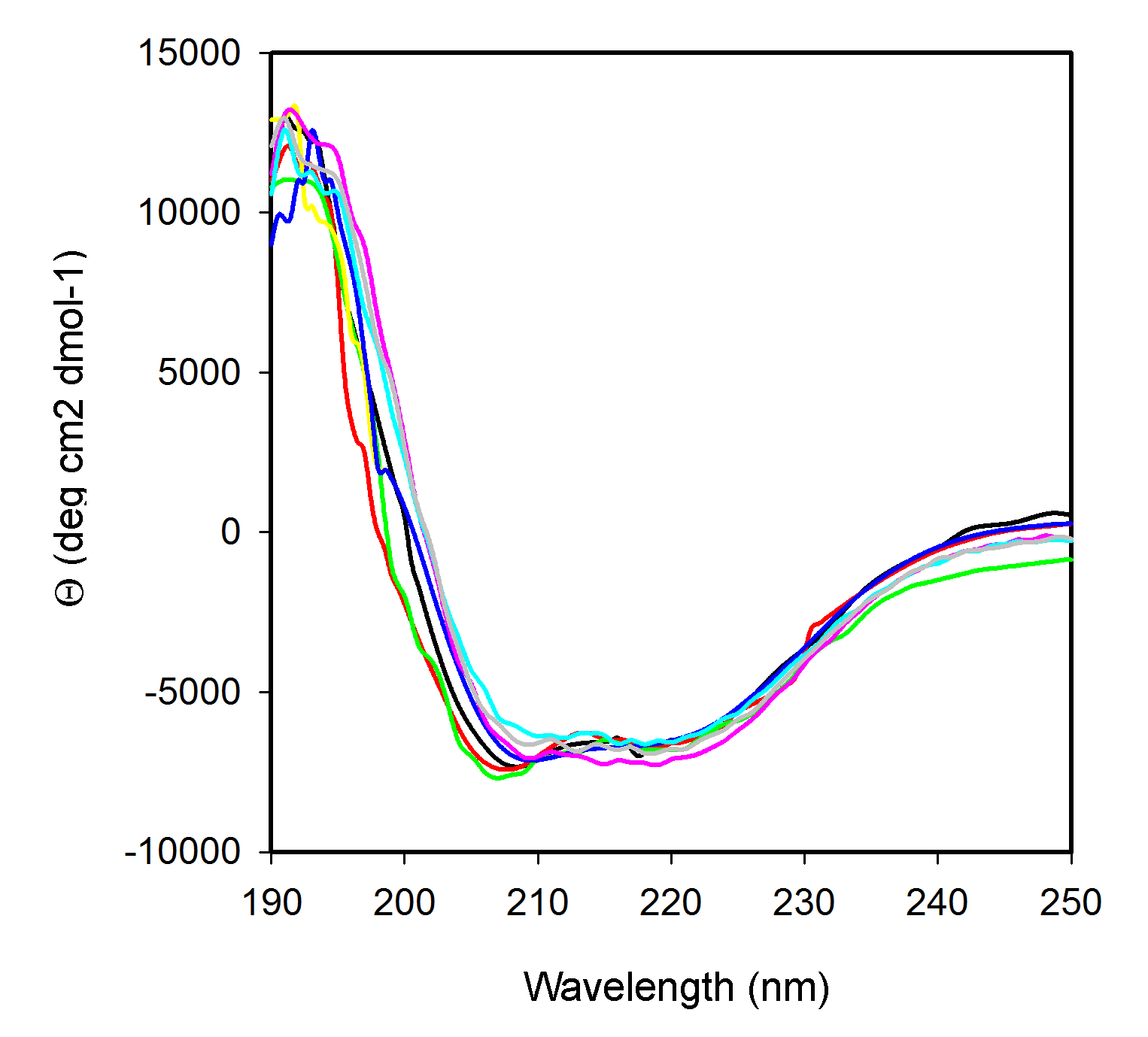


**Figure S1.** Far-UV circular dichroism spectra of the wild type and mutant CepI recombinant proteins. Black line, wild type; red line, E29Q; green line, E40E; yellow line, S41A; blue line, S41R; purple line Q46R; light blue line S147L; grey line S147R.

**Supplementary references**

1. Soding, J., Biegert, A., & Lupas, A. N. The HHpred interactive server for protein homology detection and structure prediction. *Nucleic Acids Res.* **33**, W244-248 (2005).
2. Eswar, N., *et al*. Comparative protein structure modeling using Modeller. *Curr. Protoc. Bioinformatics Chapter* **5**, Unit 5.6 (2006).
3. Adams, P. D., *et al.* PHENIX: a comprehensive Python-based system for macromolecular structure solution. *Acta Crystallogr. D Biol. Crystallogr.* **66**, 213-221 (2010).
4. Laskowski, R. A. PDBsum: summaries and analyses of PDB structures. *Nucleic Acids* *Res.* **29**, 221-222 (2001).
5. Benkert, P., Kunzli, M., & Schwede, T. QMEAN server for protein model quality estimation. *Nucleic Acids Res.* **37**, W510-514 (2009).
6. Krissinel, E., & Henrick, K.  Secondary-structure matching (SSM), a new tool for fast protein structure alignment in three dimensions. *Acta Crystallogr D Biol Crystallogr* **60**, 2256–2268. (2004).
7. The PyMOL Molecular Graphics System, Version 1.8 Schrödinger, LLC. www.pymol.org
8. Grosdidier, A., Zoete, V. & Michielin, O. SwissDock, a protein-small molecule docking web service based on EADock DSS. *Nucleic Acids Res.* **39**, W270-277 (2011).
9. Madadkar-Sobhani, A. & Guallar, V. PELE web server: atomistic study of biomolecular systems at your fingertips. *Nucleic Acids Res.* **41**, W322-328 (2013).
